# Galectin 13 (PP13) Facilitates Remodeling and Structural Stabilization of Maternal Vessels during Pregnancy

**DOI:** 10.3390/ijms20133192

**Published:** 2019-06-29

**Authors:** Marei Sammar, Tijana Drobnjak, Maurizio Mandala, Sveinbjörn Gizurarson, Berthold Huppertz, Hamutal Meiri

**Affiliations:** 1 Ephraim Katzir Department of Biotechnology Engineering, ORT Braude College, 2161002 Karmiel, Israel; 2 Faculty of Pharmaceutical Sciences, School of Health Science, University of Iceland, 107 Reykjavik, Iceland; 3 Department of Biology, Ecology and Earth Sciences, University of Calabria, 87030 Rende, Italy; 4 Department of Cell Biology, Histology and Embryology, Gottfried Schatz Research Center, Medical University of Graz, 8010 Graz, Austria; 5 Hylabs Ltd., Rehovot, 7670606 and TeleMarpe Ltd., 6908742 Tel Aviv, Israel

**Keywords:** Placental protein 13, Gal 10, Gal 13, Gal 14, Gal 16, preeclampsia, FGR, polymorphism, risk prediction, biomarkers, eNOS

## Abstract

Galectins regulate cell growth, proliferation, differentiation, apoptosis, signal transduction, mRNA splicing, and interactions with the extracellular matrix. Here we focus on the galectins in the reproductive system, particularly on a group of six galectins that first appears in anthropoid primates in conjunction with the evolution of highly invasive placentation and long gestation. Of these six, placental protein 13 (PP13, galectin 13) interacts with glycoproteins and glycolipids to enable successful pregnancy. PP13 is related to the development of a major obstetric syndrome, preeclampsia, a life-threatening complication of pregnancy which affects ten million pregnant women globally. Preeclampsia is characterized by hypertension, proteinuria, and organ failure, and is often accompanied by fetal loss and major newborn disabilities. PP13 facilitates the expansion of uterine arteries and veins during pregnancy in an endothelial cell-dependent manner, via the eNOS and prostaglandin signaling pathways. PP13 acts through its carbohydrate recognition domain that binds to sugar residues of extracellular and connective tissue molecules, thus inducing structural stabilization of vessel expansion. Further, decidual PP13 aggregates may serve as a decoy that induces white blood cell apoptosis, contributing to the mother’s immune tolerance to pregnancy. Lower first trimester PP13 level is one of the biomarkers to predict the subsequent risk to develop preeclampsia, while its molecular mutations/polymorphisms that are associated with reduced PP13 expression are accompanied by higher rates of preeclampsia We propose a targeted PP13 replenishing therapy to fight preeclampsia in carriers of these mutations.

## 1. Galectins

Galectins are a class of carbohydrate binding proteins with high affinity to β-galactoside sugars that bind to them via their N- or- O-linked glycosylation [1,2]. They share primary structural homology in their carbohydrate-recognition domains (CRDs) included in a canonical sequence of ~130 amino acid backbone. They are synthesized as cytosolic proteins and reside in the cytosol or nucleus for much of their lifetime [3]. They form a β-sandwich [4,5] consisting of five or six anti-parallel β-sheet strands [6], forming a shallow groove for holding a disaccharide or oligosaccharide. Eight amino acids form the CRD motif within this groove to mediate non-covalent binding. Additional amino acids enhance the specific interaction [5,6,7,8,9]. High affinity to the ABO blood groups is responsible for their hemagglutinin activity [10,11]. The galectins are classified into three categories [2]: (1) the prototype homo-dimers (gals 1, 2, 5, 7, 13–17, 19, 20), (2) the “tandem-repeat dimers” (gals 4, 6, 8, 9, 12) with short linkers, and (3) chimera-lectin (gal 3), with a C-terminal CRD and an N-terminal non-lectin for multimerization [12,13,14,15]. The multi-valent interaction facilitates crosslinking of signaling pathways, the formation of cell surface lattices, and endocytosis at the cell surface or in intracellular locations [16,17]. 

Today, we know of 20 members of the galectin family that interact with a plethora of molecules involved in inflammation, immune responses, cell trafficking, apoptosis, autophagy, trans-membrane signaling, and interactions with cytosolic and nuclear targets, nuclear transcription, gene expression, or mRNA splicing [18,19,20,21]. Galectins are able to translocate from intra- to extracellular compartments, and back. They affect signal transduction and apoptosis, growth, fibrosis, aggregation, adhesion, and cancer metastasis [22,23,24,25,26,27]. Hence, galectins are incorporated in the development of new therapeutics [22,27,28,29], and some are already in clinical development stages (https://galecto.com/ [30]).

## 2. The Placental Galectins

A variety of galectins are expressed in the reproductive system. They are pleiotropic regulators of key functions in the reproductive tract. Gal-1 and Gal-3 are involved in regulating signaling pathways at the feto-maternal interface [31,32] and are expressed in the endometrium and the decidua. The tandem repeat of Gal-8 acts through spliced variants in various reproductive tissues [1,2,3,4,5,6,7,8,9,10,11,12,13,14,15,16,17,18,19,20,21]. Gal-9 is abundant via its three encoding genes [33]. In this respect, it is worth mentioning that the Gal 10 protein is also expressed by white blood cells (WBC), while its mRNA is exclusively expressed in bone marrow tissues. However, the WBC reach the reproductive system and influence this system during the process of pregnancy development, especially via generating an immune response against foreign (paternal) genes of the fetus and placenta [34,35]. Other galectins in the reproductive tract such as Gal-13 participate in trophoblast invasion into the decidua, spiral artery remodeling, and immune tolerance of maternal tissues to pregnancy [2]. 

Here we focus on a placental cluster of six galectins in anthropoid primates in the context of evolution of the highly invasive placentation and long gestation [31]. The expression of these galectins in the placental syncytiotrophoblast is altered in preeclampsia and early fetal growth restriction (FGR) [32]. Three of them, Gal-13, Gal-14, and Gal-16 are uniquely expressed in the placenta, indicating the massive differentiation effort dedicated by nature for assuring the establishment and maintenance of pregnancy in eutherian mammals [36]. 

## 3. Galectin 13

### 3.1. The PP13 Protein and its mRNA

Galectin 13 (Gal-13), also known as LGALS13 and placental protein 13 (PP13), is the most studied galectin of the anthropoid primates. As one of a six cluster primate genes, it is located on chromosome 19q13 [32], and is one of 56 known placental proteins. It was first isolated from human term placenta in 1983 and characterized by Bohn et al. [37]. Normal term placenta has approximately 2.5 mg of PP13, and, according to Bohn, PP13 represents ~7% of the total placental proteins. PP13 shows structural and functional homologies to the ß-galactoside-binding lectins [1], with high homology to the other members of the cluster in their CRD [31,32,38,39]. Although so far no specific individual receptor for PP13 (in the classical sense) has been identified, affinity chromatography and mass spectroscopy determined high affinity binding of PP13 to annexin IIa, a member of Ca2+ and phospholipid binding proteins of the extracellular matrix, and to beta/gamma actin in the cytoplasm [10,40]. PP13 has high affinity to sugar residues, especially to N-acetyl glucose amine, fucose, and N-acetyl galactose amine [10]. It also binds sugar residues of the B and AB antigen of the ABO blood groups [11], a binding that regulates the availability of free PP13 in the blood of pregnant women. This binding has been found to influence the risk assessment and preeclampsia prediction of PP13 [41], as will be further detailed below. 

PP13 is expressed from a very early stage of pregnancy, and can be detected in the maternal blood already at week five of gestation [42], or 3 weeks after embryo return in IVF (Meiri, unpublished results). Immunohistochemistry and RNA hybridization studies have pointed to its predominant localization in the placental syncytiotrophoblast layer, placental blood vessels, and specific sites within the placental bed [31,42,43]. Early studies by Than et al. indicated its presence in the syncytiotrophoblast [10]. PP13 is detected in the cytoplasm and mainly along the apical plasma membrane of the syncytiotrophoblast [42,43]. It can also be detected in their nuclei, at least during very early gestation [43]. In cases of oxidative stress, strong staining for PP13 appears in the increasingly appearing syncytiotrophoblast microparticles (STBM, or necrotic bodies) [42,44]. A process of aponecrosis is accompanied by placental shedding of STBM during preeclampsia [42,45]. 

### 3.2. Insights on the Gene and Protein Structures

**Figure 1:**
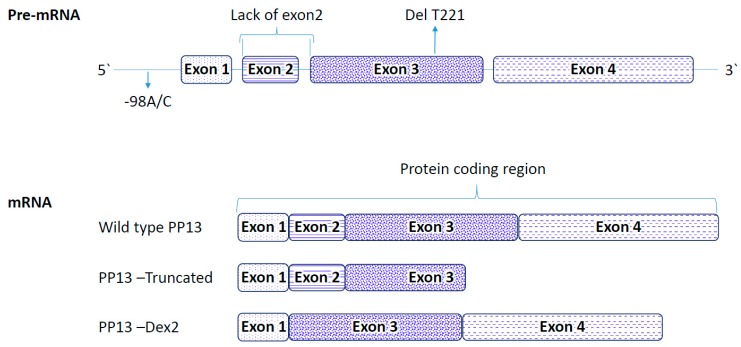
Schematic diagram of the LGALS13 gene and its mRNA variants. Top—The exons and introns are marked by boxes and lines, respectively. Lower panels represent the mRNA and the protein coding region. The wild type Gal-13 (PP13) consists of four full exons. The truncated Gal-13 variant delT_221_ is missing part of exon 3 and the full exon 4, while the Dex-2 variant is missing exon 2. The two variants—the truncated delT_221_ variant and the spliced variant Dex-2 are both naturally occurring variants along with the promoter polymorphic variant of -98 A/C.

The LGALS13 gene encodes for PP13, and is comprised of a long promoter region at the 5 prime end followed by four exons: E1 (60 bp), E2 (72 bp), E3 (211 bp), and E4 (251 bp) spaced by introns (Figure 1). Intronic regions vary between 499 bp and 1834 bp in length. Exon 4 and part of exon 3 of the LGALS13 gene exclusively code for the entire amino acids included in the CRD domain [6,10,38,46]. 

The open reading frame of PP13 encodes for 139 amino acids [10,46]. The calculated molecular weight of the monomer is ~ 16.12 kDa. In-vitro studies have shown that its expression is up-regulated by the binding of the TFAP2A transcription factor [32]. Other studies pointed to the link between PP13 expression and human chorionic gonadotropin (hCG) [47] that drives the fusion of villous cytotrophoblasts with the overlying syncytiotrophoblast [48]. Indeed, fusion of differentiating trophoblasts to form the syncytiotrophoblast is accompanied by increased PP13 expression. Fusion also increases PP13 expression in the trophoblast-derived BeWo cell line [47,49].

We engineered several recombinant PP13 variants. Initially, a Histidine-tag (His-PP13) variant was constructed, produced in E. coli, purified, and characterized [10,38]. The resultant His-PP13 fails to dimerize via disulfide bonds since the His-tag prohibits one of its cysteine SH residues from forming a dimer. The molecular conformation of such a monomeric state of PP13 prohibits the formation of the naturally occurring homodimer, and this variant tends to form a long chain of *head-to-tail* linked oligomers, which are characterized by low stability in solutions. Treatment of the His-PP13 variant with the reducing agent dithiothreitol (DTT) keeps the protein in a monomeric form, prohibiting the formation of long chain oligomers. This monomeric form exhibits long stability in solution, and in the presence of DTT lyophilized His-tag PP13 has an estimated shelf-life of 12 years or longer [50]. The second recombinant PP13 variant lacks the histidine tag (rPP13) and is expressed in E. coli [38,51]. The resultant protein was isolated from the inclusion bodies as a monomer that spontaneously homo-dimerizes to form a 32 kDa protein that is very stable in aqueous solutions. Further aggregation to trimers and tetramers is marginal [46]). 

### 3.3. PP13 Secretion from the Placenta

**Figure 2:**
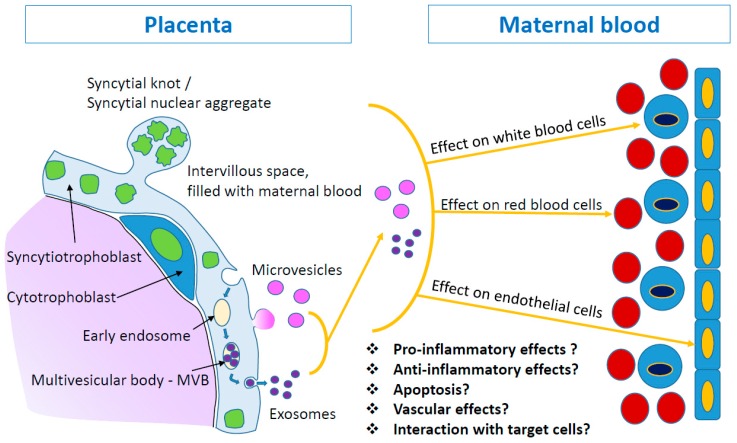
PP13 release from placental syncytiotrophoblast. Extracellular vesicles are cell-derived membrane particles, including exosomes (30–200 nm), microvesicles (100–1000 nm), and apoptotic bodies (>1000 nm). They are released from the placental syncytiotrophoblast layer. During normal turnover, the syncytiotrophoblast releases late-apoptotic syncytial knots (1–5 μm) as large corpuscular structure into the maternal blood. At the same time, microvesicles and exosomes are released and can pass through capillary blood vessels. PP13 cargo of microvesicles and exosomes appears on both types of these extracellular vesicles, on the surface and inside the vesicles. These vesicles may interact with various cell types (red and white blood cells or endothelial cells) and convey different messages to the maternal body.

Lacking a signal sequence for transmembrane transport [6], it was estimated that the release of PP13 is accomplished in a manner typical to other galectins, namely via the liberation of extracellular vesicles [12,52,53] (Figure 2). A release of un-packed protein via co-transfer with carrier proteins or endosomes was also suggested to be a calcium dependent mechanism [54,55]. In fact, it has been shown that the PP13 release from immortalized placental cells (BeWo cells) is significantly augmented with the use of a calcium ionophore [44]. Like other galectins, PP13 can re-enter cells by endocytosis via recycling of endocytic vesicles [56]. 

Sammar et al. [52] discovered a novel pathway for PP13 secretion that may be most relevant to the protein level in maternal blood. PP13 liberation is executed through the release of extracellular vesicles (EVs), mainly microvesicles and exosomes, carrying PP13 on the surface of EVs and/or inside them [52]. The microvesicles and exosomes that carry the PP13 cargo communicate with maternal organs to influence their response, both during normal and complicated pregnancies. Evidence has been obtained for the potential interaction of PP13 in such extracellular vesicles with red and white blood cells, as well as the endothelium (Figure 2). 

### 3.4. PP13 and Preeclampsia

Preeclampsia, a severe life-threatening complication of pregnancy characterized by hypertension, proteinuria and organ failure [57,58,59,60] is mainly attributed to impaired placentation [61,62]. It affects ten million pregnant women globally, and is often accompanied by fetal loss and major newborn disabilities ([63]—www.preeclampsia.org). The hunting for serum markers to predict the risk to develop this pregnancy complication was a major challenge in the first decade of the 21st century [64]. We explored the potential use of PP13 as a biomarker for predicting the risk to develop preeclampsia. The availability of the purified native and recombinant PP13 have stimulated the generation of various poly- and monoclonal antibodies, followed by the development of an ELISA immune-diagnostic kit [50]. With these tools in hands, a comparative analysis of PP13 levels in maternal blood was conducted in multiple studies [65,66,67,68]. 

The studies have shown reduced concentrations of maternal blood PP13 in the first trimester in pregnancies that subsequently developed early, preterm and term preeclampsia with and without fetal growth restriction (FGR) [66,69,70,71,72,73,74,75,76,77,78]. Longitudinal studies have shown that in preeclampsia there is a sharp increase of PP13 between the first to the third trimester with the slope of change predicting the severity of the subsequent complication [42,75]. Such PP13 increase also predicts severe hemorrhage after delivery [79]. Interestingly, in twin pregnancies that subsequently develop preeclampsia, the level is very high already in the first trimester, indicating accelerated processes of impaired placentation in multiple pregnancy, corresponding with their higher frequency of the disorder [68].

In studies where term placentas were obtained after delivery, the mRNA levels of PP13 were 3.5-fold lower in women who developed PE and the related HELLP syndrome [45,65]. Additional studies have shown that the reduced PP13 mRNA can be determined already in the first trimester in patients who subsequently developed preeclampsia [80,81,82]. Unlike the protein level that tends to increase near the time of disease, low PP13 mRNA was detected throughout pregnancy. It was subsequently discovered by in-vitro placental explant studies that in a normal pregnancy the release of PP13 from a single placental villus is decreased from the first to the third trimester. During the first trimester of normal pregnancy the level of PP13 in maternal blood increases from 200–300 pg/mL to 400–600 pg/mL [42] due to the increase in the total number of villi during pregnancy [83]. In contrast, villi of preeclamptic placentas showed an elevated PP13 release at the time of disease [83]. It is estimated that aponecrotic release of PP13 from the large number of damaged villi accounts for the sharp slope of the PP13 level in maternal blood during the etiology of preeclampsia [36]. 

In a meta-analysis of 18 studies that investigated maternal blood levels of PP13 during the first trimester, reduced PP13 levels were found in women who subsequently developed preeclampsia about 20 weeks later. If evaluated in the first trimester as a single biomarker PP13 provided 83% detection rate for 10% false positive rate for early preeclampsia (<34 weeks), 66% for preterm preeclampsia (<37 weeks), and 47% for all cases of preeclampsia [66]. PP13 combined with first trimester Doppler pulsatility index of the blood flow through the maternal uterine arteries and the use of additional markers provides higher detection rates of preeclampsia in the first trimester [66,84]. 

### 3.5. PP13 Polymorphism and Preeclampsia

Polymorphic variants of PP13 have been identified [38,66], and three of them are important indicators of a high risk to develop preeclampsia:

(1) The “truncated” variant is a deletion of thymidine in position 221 of the open reading frame of exon 3 [85,86]. It was discovered among black and colored pregnant women in a Cohort of Cape Town, South African cohort [85]. It is associated with the development of an earlier stop codon coupled to a shorter PP13 variant (“truncated” or “delT221”) [38]. The shorter delT221 variant is lacking the entire exon 4 and part of exon 3 [38] (Figure 1). Hence, delT221 is missing 2 of the amino-acids involved in the carbohydrate recognition domain (CRD), and two additional amino acids supporting carbohydrate binding [6,31]. Having this mutation in a heterozygous form is an effective predictor of severe early preeclampsia with 89% positive predictive value. Treatment of human leukocytes derived of the maternal decidua with the wild type of recombinant PP13 but not with the truncated PP13 induced apoptosis [31,43]. From this data it was speculated that one role of PP13 in pregnancy is to render the mother immune-tolerant to pregnancy. The immune tolerance is reached by binding of PP13 via the CRD to glycoproteins and glycolipids. Indeed, pregnancies carrying the homozygous DelT221 mutation are rejected by the mother and are not viable [86]. 

(2) The promoter variant. The -98 (A/C) promoter genotype displays three genotypes: the “A/A” genotype (homozygous to the adenosine nucleotide), the “C/C” genotype (homozygous to cytosine), or the “A/C” genotype (heterozygous form). In a South African as well as a London cohort of pregnant women, the A/A genotype was found to be associated with decreased expression of PP13 compared to the level of PP13 expression with either A/C or C/C genotypes in the -98 position [51,87]. The reduced expression was contributed in part by the impaired ability of the transcription factor TFAP2A to induce PP13 expression with the A/A genotype [51]. Accordingly, carriers of the A/A variant had an adjusted odds ratio of 3.68 to develop preeclampsia, while the C/C or the A/C genotypes rendered protection from developing preeclampsia. Combining the A/A genotype as a risk factor together with black ethnicity, history of previous preeclampsia, obesity (BMI > 37), and being at advanced maternal age provided an adjusted odds ratio of 14.0 and 7.0, respectively, for developing term or all preeclampsia cases [51]. 

(3) The Dex-2 variant. Recently, we were able to molecularly engineer a third molecular variant of PP13 that was denoted Dex-2. This mutant completely lacks the second exon (Figure 1). This additional natural PP13 variant was initially isolated in Israel while cloning PP13 DNA from a genomic library. The mutant clones were isolated from a placenta obtained after delivery from a woman with preeclampsia combined with FGR [55]. Burger et al. [55] have shown that PP13 which was isolated from a placenta of preeclampsia with FGR was inferior in inducing the liberation of free fatty acids from trophoblast membranes, and in causing the elevated release of prostaglandins. Further analysis of this mutant is warranted.

In summary, there may well be a link between reduced levels of PP13 during the first trimester of human pregnancy and the elevated risk for a subsequent development of preeclampsia. Preeclampsia patients may be a target population to evaluate if nourishing with the wild-type, full length PP13 can be used as a therapeutic tool to fight preeclampsia.

### 3.6. PP13 and Immune Tolerance

The syncytiotrophoblast secretes/releases PP13 from the first trimester and the protein reaches the decidua either via diffusion or via the maternal circulation, coinciding with the time of early trophoblast invasion. Kliman et al. [43] have shown the formation of PP13 aggregates closer to areas with increased apoptosis of various maternal immune cells. Killing these cells could enable us to promote extravillous trophoblast invasion of the uterine wall. In this manner, PP13 might serve to establish a decoy inflammatory response, sequestering maternal immune cells away from the site of extravillous trophoblast invading other sites of the uterine wall [31,39,43]. Accordingly, it was proposed that PP13 contributes to the immune tolerance of the mother to the invading trophoblasts. Having low levels of PP13 and/or having a mutated variant may decrease the level of PP13 secretion, thereby contributing to impaired placentation. 

### 3.7. PP13 Replenishing Studies in Animals

The uteroplacental circulation undergoes massive changes during pregnancy, resulting in a vascular system that is directing 20% of the total cardiac output to the uterine vascular bed. This results in more than a ten-fold increase in blood flow over the level present in the non-pregnant state [88]. Since in normal pregnancy there is only a small drop in blood pressure, it is necessary to gain uterine hemodynamic changes by uterine blood vessel expansion and reduced uterine vascular resistance [89]. In pregnancy, extravillous trophoblasts invade all types of luminal structures in the placental bed [90,91]. One of their major targets are spiral arteries and their adjacent stroma. The endoarterial trophoblast subpopulation [92] replaces and reorganizes the vascular smooth muscle and endothelial layers, resulting in the formation of low-resistance vessels that can accommodate a highly increased blood volume flowing towards the placenta [93]. These altered vessels are almost independent of maternal vasoconstriction through a lack of smooth muscle cells [89,94].

Through the invasive processes of the extravillous trophoblast, the vessels towards the intervillous space of the placenta (spiral arteries) and those draining blood back into the maternal system (uteroplacental veins) are connected to the placenta, resulting in a placental blood flow to sufficiently supply the placenta and the growing fetus with nutrients and oxygen [89,91]. This hemochorial type of placentation is present in mammals such as humans, higher order primates, rabbits, guinea pigs, mice, and rats [95,96,97]. At term there are around 200 spiral arteries opening towards the intervillous space, while the blood flow in the uterine artery is increased in volume with reduced velocity [96]. Impaired trophoblast invasion into spiral arteries results in higher blood flow velocities into the intervillous space of the placenta and thus damage of the fragile villous trees. [98,99,100,101]. 

PP13 appears to have an important role in these hemodynamic changes by facilitating expansion of the uterine vascular system during pregnancy to accommodate the increase of blood flow through the uterus and thus the placenta during pregnancy. The following in vivo data have been obtained using PP13 administration in different animal models: 

• Initially, a single PP13 dosage injected intravenously into gravid rats and rabbits resulted in a reversible ~30% reduction in blood pressure [102]. 

• In a second set of experiments, peristaltic pumps were implanted into gravid rats for a slow release of PP13 for 4 to 7 days from day 15 [102], or from day 8 of pregnancy [103]. rPP13 (compared to saline control) reversibly reduced blood pressure until the pumps released all their content. At delivery, 5 to 7 days after the active release of PP13 was over, treated animals had larger placentas and pups. Both the wild type rPP13 and the truncated variant DelT221 were effective in reducing blood pressure, but the truncated variant failed to sustain uterine artery expansion until the time of delivery [103].

• Isolated uterine mesenteric arteries from both mid-pregnant and non-pregnant rats were placed in arteriographs to measure their diameters and pressure in response to drug perfusion [104]. Uterine arteries of both pregnant and non-pregnant rats were dilated in a dose dependent manner with increasing concentrations of PP13. Half-maximal vasodilation of isolated arteries (EC50) was achieved at a concentration of 1pM PP13 (blood level of pregnant women). The effect was mediated by the endothelial layer, since stripping the vessels off the endothelial layer prohibited blood vessel expansion by PP13. Pharmacological analysis of the signaling pathways revealed that the vasodilation was mediated through signaling of the endothelial nitric oxide synthase (eNOS) and prostaglandin type 2 pathways [104]. 

• An additional study was performed with non-pregnant rats. Again, surgically implanted pumps released a constant dose of PP13 (rPP13 or His-PP13 variants) or saline over seven days. Some animals were sacrificed immediately after the end of PP13 release (on day 7), while others were sacrificed 6 days later (day 13) to compare the short and long-term impacts of PP13 on vessel growth and size. Both uterine veins and arteries were significantly expanded by rPP13 with a more pronounced effect after 13 days compared to the corresponding vessels after seven days. The long-term effect of treatment by rPP13 was more pronounced in the veins compared to the corresponding arteries. His-PP13 also expanded the blood vessels but the effect remained similar between 7 and 13 days, most likely since His-tag PP13 has only a monomeric form. This molecular variant does not turn into the natural configuration of a homo-dimer. It is estimated that to exert the structurally stable vascular expansion that is developed with the non His-tag protein, a molecular variant that forms a homo-dimer is required [105].

**Figure 3:**
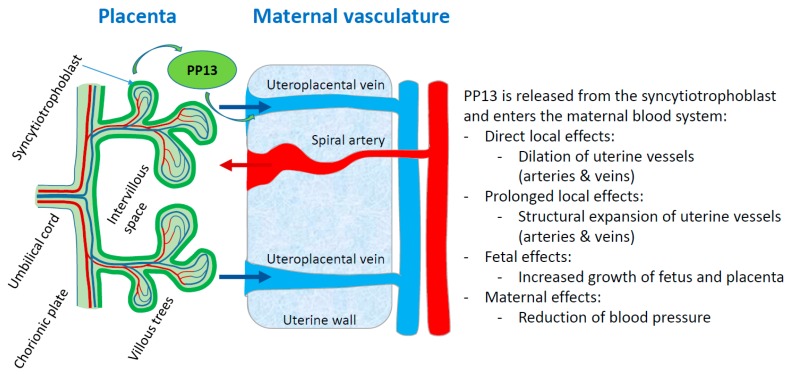
PP13 priming of maternal blood vessels. The scheme displays a comprehensive model of the PP13 effects on the vascular system of the mother. PP13 is released from the syncytiotrophoblast and enters the maternal blood system where it has different effects. The red arrow shows flow of maternal blood into the placenta via invaded spiral arteries, while the blue arrows indicate flow of maternal blood back from the placenta into the maternal vascular system via invaded uterine veins.

In conclusion, PP13 appears to play a key role in the remodeling of uterine arteries and veins during pregnancy, facilitating the adjustment of blood flow to and from the placenta. This way, PP13 adapts the uterus to provide increased but slower blood flow towards the placenta and back into the maternal system, necessary for normal pregnancy. PP13 acts via the NO and prostaglandin signaling pathways to provide oxygen and nutrients to the growing fetus (Figure 3).

### 3.8. Modeling the Role of PP13 in Pregnancy

**Figure 4:**
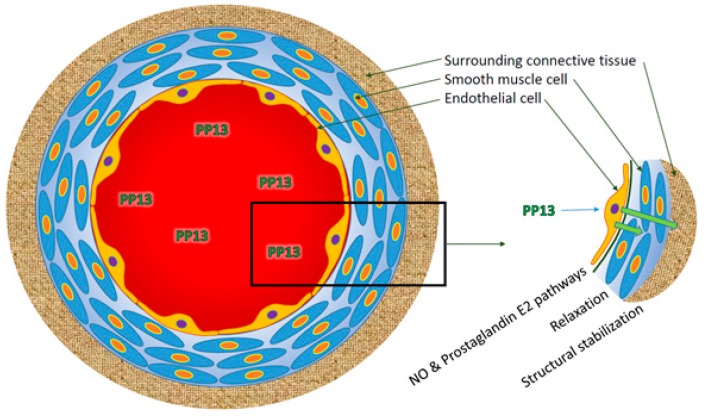
The effect of PP13 on the various layers of blood vessels. PP13 acts on the endothelial layer of the blood vessels and causes vasodilation by muscle relaxation through the signaling pathways of eNOS and prostaglandin 2. Further, PP13 causes stabilization of the surrounding connective tissues. Molecularly, this process requires the carbohydrate recognition domain to cross link between PP13 and molecules on the surface of the connective tissue and extracellular matrix.

The PP13 molecule primes blood vessel expansion to adapt the uterine vascular system to supply oxygen and nutrients to the growing fetus. The effect supports the development of larger placentas and pups, as shown in a rat model [103]. The effect involves a chain of reactions, starting from a physiological effect that involves the endothelial layer through the e-NOS and prostaglandin signaling pathways [103], and continuing through structural stabilization of the surrounding components of the connective tissue around the blood vessels. Connective tissue stabilization requires the CRD component of the PP13 molecules that crosslinks between the endothelial layer and the connective tissue (Figure 4). Finally, PP13 acts as a decoy to attract maternal immune cells and thereby enabling the invasion of extravillous trophoblast into blood vessels [43].

Based on all the above we propose a targeted PP13 therapy to fight preeclampsia in patients with impaired PP13 and high risk to develop preeclampsia [106]. These patients could receive PP13 as a nourishing drug to support uterine vessel expansion and stabilization of blood supply during pregnancy. We are conducting preclinical studies and plan to evaluate the potential clinical impact testing animal models of preeclampsia to explore this hypothesis. 

Considering the multifaceted nature of preeclampsia [58,59,60], the development of PP13 as a novel biological therapy to fight preeclampsia is now evaluated in certain animal models to provide a proof of concept. Among animal models, we plan to test (1) the reduced uterine placenta perfusion (RUPP model) in rats [107], (2) the transgenic mouse model of STOX-1 [108], and (3) the Baboon uteroplacental ischemia model [109], all identified as important model systems to evaluate novel drugs to fight preeclampsia [110,111].

## 4. Multiple Galectins and Deep Placentation

In this article we focus on PP13, a member of the cluster of 6 galectins that emerged during primate evolution, and are only found in anthropoids. These species differ from their strep-sirrhine counterparts by having hemochorial placentas associated with a reduction in the number of offspring, with just one infant being common in monkeys, humans and apes. In all of these species, the newborns have relatively large brains and long gestations [112,113]. As described before, the success of pregnancy is mediated via increased blood flow to and from the placenta, which is achieved via an invasive hemochorial placentation [114,115,116]. The genetic differences between the mother and the fetal semi-allograft necessitate the development of immune tolerance to reduce the danger of fetal rejection by the mother, considering the alloantigen aspect of eutherian pregnancies [32,114]. We have provided evidence for the crucial role of galectin 13 (PP13) to render the mother immune-tolerant to sustain the hemochorial placentation during the long gestation of anthropoid primates. Interestingly, it has been pointed out that in addition to PP13, the other members of the cluster of galectins of chromosome 19 in anthropoids share high homology in their sequence and placental localization ([117] https://www.ncbi.nlm.nih.gov/kis/ortholog/29124/?scope=9526#genes-tab).

Table 1 indicates that humans have the entire cluster of which 5 are exclusively expressed in the placenta and one (Gal-10) is expressed in the bone marrow, but reaches the placenta via white blood cells, mainly eosinophils [2,31,32,39]. Orangutans, macaque, Sp. monkeys, and marmosets have four of the galectins, chimpanzees have two, and baboons, gorillas, and colobuses have only one galectin [31]. 

Interestingly, all of the above species with the exception of the baboon, have PP13, most likely reflecting that this protein may be the first to be evolved or is derived from a common ancestor, and potentially it is the most essential one for a successful intrusive pregnancy [31]. The second most frequently found is Gal-14 that appears in four species. Gal-10 and Gal 16 are expressed in three species, while Gal-17 appears in two species and Gal-20 only in one. In terms of sequence homology, all galectins have close to 98% homology in the composition and configuration of their major amino acids of the carbohydrate recognition domain, the CRD. Gal-13 and Gal-16 share 73% amino acid sequence homology, while homology between Gal-13 and Gal-14 and between Gal-13 and Gal-10 is at the level of 68% and 57%, respectively. Interestingly, human Gal-14 and baboon Gal-14 have 98% amino acid sequence homology [34,36].

According to Carter et al. [118] there are different models of placentation among apes. Yet, gorilla, chimpanzee, and human species have the deepest trophoblast invasion and their remodeling of the spiral arteries occurs deep into their inner myometrium [114,115,116,117,118,119,120]. All three have Gal-13 (PP13), while baboons with a much shallower trophoblast invasion only express Gal-14. Thus, having multiple co-expression of galectins appears to be essential for successful invasive pregnancy, in which PP13 is pivotal but may not be the only one required. The interplay between the different galectins and their composition is now under study to understand their crucial role in normal pregnancy and pregnancy complications.

## Figures and Tables

**Table 1:** Placental galectins in primates.

Species	Gal 10 Eosinophils	Gal 13 Placenta	Gal 14 Placenta	Gal 16 Placenta	Gal 17 Placenta	Gal 20 Placenta	Count	Invasion Level
**Chimpanzee**							2	3+
**Orangutan**	10A				17C		4	2+
**Baboon**							1	1+
**Human**	10A				17A 17B		5 (with 2 subtypes of Gal 17)	4+
**Gorilla**							1	3+
**Colobus**							1	1+
**Macaque**					17C		4	2+
**Marmoset**	10A 10B 10C						4 (with 3 subtypes of Gal 10)	2+
**Sp. Monkeys**	10A 10B 10C						4 (with 3 subtypes of Gal 10)	2+
**Total species #**	4	8	5	4	3	2		

The presence of placental galectins in primate placenta is provided following the analysis of the evolutionary differentiation tree [31,32,36,39,115] with the exception of Galectin 10 (Gal 10) that is generated in bone-marrow but reaches the placenta via its expression in white blood cells. The letters A, B, and C reflect isoforms of the molecules. In terms of invasion: gorilla, chimpanzee, and human species have the deepest trophoblast invasion (3+ and 4+) reaching the inner myometrium [114,115,116,117,118,119,120]. The others have a much shallower implantation (1+ or 2+). The color code indicates in which species the Gal isoforms are expressed. The numbers in “total” refer to the numbers of species in which a specific Gal isoform is expressed. The numbers in “Count” refer to the numbers of Gal isoforms expressed in a given species.

## Abbreviations

ABO blood groups	Blood group types A, B, AB and O
BP	Base pair
CRD	Carbohydrate-recognition domain
EC_50_	Effective dose for reaching 50% effect
Enos	Endothelial nitric oxide synthase
Gal 1, Gal 3, etc.	Galectin 1, galectin 3 and other galectins according to their nomenclature
FGR	Fetal growth restriction
kDa	Kilo-Dalton
pMol	Pico molar quantity (10^-12^ M)
PP13	Placental protein 13 also called gal-13 and LGALS13 (gene)
STBM	Syncytiotrophoblast microparticles
WBC	White blood cells
